# Continuity of care and advanced prostate cancer

**DOI:** 10.1002/cam4.5845

**Published:** 2023-03-23

**Authors:** Ravishankar Jayadevappa, Thomas Guzzo, Neha Vapiwala, Stanley Bruce Malkowicz, Joseph J. Gallo, Sumedha Chhatre

**Affiliations:** ^1^ Department of Medicine, Perelman School of Medicine University of Pennsylvania Philadelphia Pennsylvania USA; ^2^ Leonard Davis Institute of Health Economics University of Pennsylvania Philadelphia Pennsylvania USA; ^3^ Division of Urology, Department of Surgery, Perelman School of Medicine University of Pennsylvania Philadelphia Pennsylvania USA; ^4^ Abramson Cancer Center University of Pennsylvania Philadelphia Pennsylvania USA; ^5^ Corporal Michael J. Crescenz VAMC Philadelphia Pennsylvania USA; ^6^ Radiation Oncology, Perelman School of Medicine University of Pennsylvania Philadelphia Pennsylvania USA; ^7^ School of Public Health Johns Hopkins University Baltimore Maryland USA; ^8^ Department of Psychiatry, Perelman School of Medicine University of Pennsylvania Philadelphia Pennsylvania USA

**Keywords:** advanced prostate cancer, continuity of care, cost of care, health service use, mortality, racial disparity, SEER‐Medicare database

## Abstract

**Background:**

Continuity of care is an important element of advanced prostate cancer care due to the availability of multiple treatment options, and associated toxicity. However, the association between continuity of care and outcomes across different racial groups remains unclear.

**Objective:**

To assess the association of provider continuity of care with outcomes among Medicare fee‐for‐service beneficiaries with advanced prostate cancer and its variation by race.

**Design:**

Retrospective cohort study using Surveillance, Epidemiology, and End Results (SEER)‐Medicare data.

**Subjects:**

African American and white Medicare beneficiaries aged 66 or older, and diagnosed with advanced prostate cancer between 2000 and 2011. At least 5 years of follow‐up data for the cohort was used.

**Measures:**

Short‐term outcomes were emergency room (ER) visits, hospitalizations, and cost during acute survivorship phase (2‐year post‐diagnosis), and mortality (all‐cause and prostate cancer‐specific) during the follow‐up period. We calculated continuity of care using Continuity of Care Index (COCI) and Usual Provider Care Index (UPCI), for all visits, oncology visits, and primary care visits in acute survivorship phase. We used Poisson models for ER visits and hospitalizations, and log‐link GLM for cost. Cox model and Fine‐Gray competing risk models were used for survival analysis, weighted by propensity score. We performed similar analysis for continuity of care in the 2‐year period following acute survivorship phase.

**Results:**

One unit increase in COCI was associated with reduction in short‐term ER visits (incidence rate ratio [IRR] = 0.65, 95% confidence interval [CI] 0.64, 0.67), hospitalizations (IRR = 0.65, 95% CI 0.64, 0.67), and cost (0.64, 95% CI 0.61, 0.66) and lower hazard of long‐term mortality. Magnitude of these associations differed between African American and white patients. We observed comparable results for continuity of care in the follow‐up period.

**Conclusions:**

Continuity of care was associated with improved outcomes. The benefits of higher continuity of care were greater for African Americans, compared to white patients. Advanced prostate cancer survivorship care must integrate appropriate strategies to promote continuity of care.

## Graphical abstract

Summary of models on the interactive effects of race and continuity of care (overall) on ER visits, hospitalizations, cost, all‐cause mortality and cancer‐specific mortality, weighted by propensity score—advanced‐stage prostate cancer.

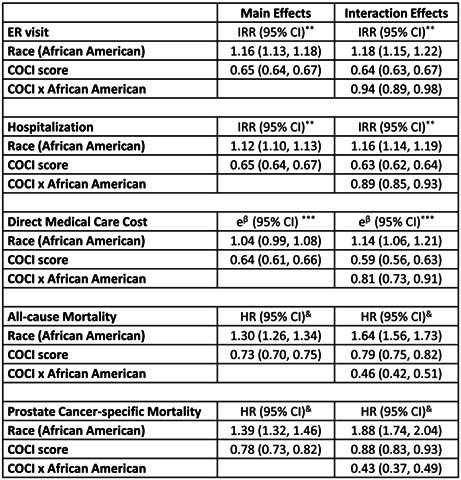

## 1 INTRODUCTION

Prostate cancer is the most commonly diagnosed cancer among men In the United States, and caring for prostate cancer places substantial burden on Medicare.^1^ Rates of advanced‐stage prostate cancer are projected to increase through 2025.^1^, ^2^ Therapeutic advances have led to improved survival for many advanced prostate cancer patients. Research has observed disparities in the quality of prostate cancer care between racial and ethnic groups.^3^, ^4^, ^5^ Disparities exist in prostate cancer treatment and process of care^3^, ^4^, ^6^, ^7^ African American men were more likely to have aggressive prostate cancer with higher comorbidity, and mortality.^3^, ^4^, ^8^, ^9^, ^10^, ^11^, ^12^ The racial and ethnic disparity in prostate cancer care and outcomes is also affected by factors that are non‐clinical in nature.^13^, ^14^ Among prostate cancer patients, cause of death was observed to vary by personal and clinical attributes.^15^ Although research has demonstrated race to be a key factor in predicting treatment and outcomes of care, whether the association between continuity of care and outcomes differs by racial and ethnic groups of advanced prostate cancer patients remains unclear.^3^, ^4^, ^13^, ^14^

Continuity of care implies responding to the health‐related needs of patients that is coordinated and without interuptions.^16^, ^17^, ^18^, ^19^, ^20^, ^21^, ^22^, ^23^ Care fragmentation is a result of duplicative services, and can lead to impaired outcomes.^19^, ^20^, ^24^, ^25^ Higher continuity of care may help lower the costs of care, improve trust, and communication between patients and clinicians, and enhance satisfaction with care.^17^, ^18^, ^23^, ^24^, ^25^, ^26^, ^27^, ^28^, ^29^, ^30^, ^31^ Continuity of care is an important element of quality of care,^16^, ^24^, ^32^ and of prostate cancer care given the different treatment alternatives, lasting effects of these treatments and the natural history of the disease that generally predisposes a patient to fragmented care.^19^, ^21^, ^24^, ^25^, ^26^ Prostate cancer treatment involves input from different providers which can lead to impaired coordination and communication, thus worsening the adverse effects associated with treatment, and cost of care.^26^, ^31^, ^33^, ^34^, ^35^ Currently, there are in excess of 3 million prostate cancer survivors who account for nearly $7 billion in annual spending. Thus, continuity of care can play an important role in improving care for prostate cancer survivors.^17^, ^18^, ^21^, ^22^, ^24^, ^35^

Several types of continuity of care measures such as Continuity of Care Index (COCI), Herfindahl–Hirschman Index (HHI), Usual Provider Care Index (UPCI), and Usual Provider Care (SECON) can be developed using administrative data. The COCI is a measure of the dispersion of visits and denotes the level to which a patient's visits over a certain length of time are with a single provider or with a group of providers.^16^ The COCI is also a measure of interpersonal continuity of care and can be used to model the ability of a healthcare system to maintain ongoing relationships between patients and healthcare providers. Medicare claims data for advanced‐stage prostate cancer facilitates calculation of COCI given that each patient can have multiple medical visits with different health care providers.^36^, ^37^ The UPCI is another widely used continuity metric.^25^ It represents the concentration of visits with a single usual provider (or with a group of usual providers) during an episode and is thus a measure of the visit density.^25^, ^37^ The HHI is a measure of market concentration, and SECON is focused on order of visits.^29^ We have used COCI and UPCI as measures of continuity of care to assess both the dispersion and density of visits.

Survivorship is defined by the American Society of Clinical Oncology as living with cancer.^38^ A large proportion of prostate cancer patients are treated in the 2‐year period after the diagnosis of prostate cancer, and may experience treatment‐related effects beyond the treatment phase. Thus, in this study, the 2‐year period after the diagnosis of prostate cancer is considered as the acute survivorship phase. The objective of our study was two‐fold. First, we assessed if continuity of care during the acute survivorship phase was associated with short‐term outcomes of emergency room (ER) visits, hospitalizations, and cost and with long‐term (up to 16 years) outcomes of all‐cause mortality and prostate cancer‐specific mortality. Next, we studied the moderating effect race may have on the association between continuity of care and outcomes in out cohort of Medicare beneficiaries with advanced‐stage prostate cancer. Our hypothesis was that greater continuity of care in the acute survivorship phase will be is associated with improved outcomes for African American, and white patients with advanced prostate cancer. Finally, we also assessed the association between continuity of care in the 2‐year period following the acute survivorship phase and short and long‐term outcomes.

## 2 METHODS

### 2.1 Data source and sample

This was a retrospective study of data from Surveillance, Epidemiology, and End Results (SEER)‐Medicare for years 2000 to 2016. The National Cancer Institute's SEER program collects data related to cancer that includes cancer incidence, treatment for cancer, and mortality from 17 SEER sites that encompass 26% of the total United States' population. Among the cancer patients from SEER registries who are of age 65 or older, majority (93%) have also been identified in Medicare.^39^ This study received approval from the local institutional review board.

### 2.2 Study cohort

The cohort comprised of African American and white, fee‐for‐service Medicare beneficiaries diagnosed with prostate cancer between 2000 and 2011. Claims in the 1 year pre‐diagnosis period are needed to assess comorbidity, and therefore, we retained patients who were aged 66 or older when diagnosed with prostate cancer. Other inclusion criteria were advanced disease stage, and at least two outpatient visits that were for evaluation and management during the acute survivorship phase. The end of study date was December 31, 2016. Thus for the patients alive as of end of study, follow‐up period was the time between diagnosis and end of study; whereas, for those deceased prior to end of study, the follow‐up ended on date of death.

### 2.3 Study variables

Key independent variable was continuity of care, and race (African American and white) was the moderator variable. Outcomes (ER visits, hospitalizations, cost, all‐cause mortality, and prostate cancer‐specific mortality) were the dependent variables, and demographic and clinical attributes were covariates.

### 2.4 Independent variables

#### 2.4.1 Continuity of Care Index

We calculated the COC index using the following formula:

$$\mathrm{COC}=\sum_{j}^{s}n_{j}^{2}-n/n\left(n-1\right)$$

where *n* is the total number of visits, ‘*n*_*j*_’ is the number of visits to provider 1, and ‘*s*’ is the number of providers. The COCI value ranges between 0 and 1. Higher COCI is indicative of higher continuity of care. When s is equal to one, or in other words, when all of the visits are with the same provider, the COCI is equal to one and denotes maximum continuity.^36^, ^37^

#### 2.4.2 Usual Provider Continuity Index

The UPCI was calculated as *n*_*i*_/*N*, where, *n*_*i*_ is the number of visits to main provider, and *N* is the total number of visits. If a single ‘main provider’ is not identified, the most frequent provider is considered to be the ‘main provider’ and is represented as: *n*_*i*_ = max (*n*_1_, *n*_2_, *n*_3_…), where *n*_1_ is the number of visits to provider 1, *n*_2_ is the number of visits to provider 2, *n*_3_ is the number of visits to provider 3, and so on. The UPCI ranges between 0 and 1, and the higher value implies higher continuity of care.^25^, ^37^, ^40^

#### 2.4.3 Identification of outpatient visits

We used outpatient and provider service claims to identify visits for the purpose of evaluation and management during the acute survivorship phase (Table S1).^18^, ^22^, ^34^ Physician specialty data from American Medical Association was linked using Unique Provider Identification Number. From the total visits, we separated visits with oncology provider, and visits with primary care provider (Table S2).^18^, ^22^, ^34^ The COCI and UPCI was computed for total visits, for oncology visits and for primary care visits.

#### 2.4.4 Race

We obtained data on race (African American and white) from the SEER‐Medicare's Patient Entitlement and Diagnosis Summary File (PEDSF).

#### 2.4.5 Demographic and clinical characteristics

Data on demographic variables (age at diagnosis, marital status, and census tract poverty index), were obtained from PEDSF. Clinical attributes were comorbidity measured as Charlson comorbidity score, cancer grade, and treatment for prostate cancer. To calculate Charlson comorbidity score, we used claims for hospitalizations, outpatient, and provider visits in the 1‐year pre‐prostate cancer diagnosis period.^30^ Treatment was extracted from both PEDSF and Medicare claims.

#### 2.4.6 Outcomes

Short‐term outcomes were ER visits, hospitalizations, and cost in the acute survivorship phase. We used outpatient claims to determine ER visits that did not result in hospitalizations. We used MEDPAR files of SEER‐Medicare to identify hospitalizations. In this study, we operationalized cost as reimbursements made by Medicare. Total cost included reimbursements made for hospitalizations, outpatient services, and provider services.

The long‐term outcomes were mortality (all‐cause and prostate cancer‐specific) assessed over the entire study period (up to 17 years post‐prostate cancer diagnosis). We determined vital status using data from both SEER and Medicare. Patients who were alive as of end of study (December 31, 2016) were censored.

### 2.5 Statistical analysis

We first examined the distribution of all variables in our cohort of advanced prostate cancer patients by race. Our main analysis assessed the association of continuity of care (operationalized as COCI and UPCI), with short‐term and long‐term outcomes. We also conducted separate analysis for COCI (and UPCI) for all visits, for oncology visits, and for primary care visits. We used Poisson regression for analyzing the short‐term outcomes of ER visits and hospitalizations. For assessing short‐term cost, we used generalized linear models (GLMs) with log‐link and gamma distribution. For survival analysis related to all‐cause mortality, we used Cox proportional hazard model. For competing risk of prostate cancer‐specific mortality, we used Fine and Gray model. We also calculated COCI in the 2‐year period following the acute survivorship phase and studied its association with short‐term and long‐term outcomes.

We evaluated two sets of models for each outcome. The first set of models was to assess the main effects of continuity of care (operationalized as COCI and UPCI). In the second set, interaction of race and continuity of care was introduced in the model. The interaction term was used to evaluate if the association between continuity of care and outcomes differed across African American and white patients. The results of Poisson models were reported as incidence rate ratios (IRRs) and 95% confidence interval [CI]. The GLM model result was in the form of exponentiated beta estimates (e^*β*^) and 95% CI. Survival models yielded hazard ratios (HRs) and 95% CIs.

#### 2.5.1 Propensity score

Prostate cancer treatment assignment is non‐random, therefore, we adopted the propensity score technique to address the observed confounders.^28^ We used multi‐nominal logistic regression to estimate the probability of being treated with a specific type of prostate cancer treatment after adjusting for socio‐demographic characteristics (age, race, marital status, and census poverty tract index), and clinical characteristics (grade and comorbidity). All analytical models were weighted by the inverse of the probability (propensity) score.

## 3 RESULTS

### 3.1 Descriptive statistics

Between 2000 and 2011, there were 611,832 new cases of prostate cancer. After applying study criteria 19,721 advanced‐stage patients were retained In the study (Figure S1). In Table 1, we present the comparison of baseline socio‐demographic and clinical characteristics of the cohort by race. African American patients were younger compared to their white counterparts (mean age 73.4 years vs. 74.0 years). Marital status, Census poverty index, comorbidity, and treatment type differed between African American and white patients. Continuity of care (for all visits and for visits with oncology provider) was higher for African Americans compared to their white counterparts (mean COCI 0.45 vs. 0.43, *p* < 0.0001; and 0.77 vs. 0.75, *p* < 0.0001, respectively).

**TABLE 1:** Descriptive statistics for socio‐demographic and clinical characteristics in overall sample, and by race—advanced‐stage prostate cancer.

	All participants, No. (%)	White, No. (%)	African American, No. (%)	*p* value^a^
	(*N* = 19, 721)	(*n* = 17,389)	(*n* = 2332)	
Age in years (mean ± SD)^b^	73.8 ± 6.3	74.0 ± 6.3	73.4 ± 6.1	0.0004
Marital status, *n* (%)^c^				
Married	14,225 (72.1)	13,051 (75.1)	1174 (50.4)	<0.0001
Census poverty index, *n* (%)^c^				
0% to <5% poverty	5998 (30.4)	5819 (34.5)	179 (7.7)	<0.0001
5% to <10% poverty	5650 (28.7)	5386 (30.9)	264 (11.3)	
10% to <20% poverty	4937 (25.0)	4317 (24.8)	620 (26.6)	
20% to 100% poverty	3136 (15.9)	1867 (10.7)	1269 (54.4)	
Comorbidity, *n* (%)^c^				
0	13,824 (70.1)	12,321 (70.9)	1503 (64.5)	<0.0001
1–2	3726 (18.9)	3272 (18.8)	454 (19.5)	
≥3	2171 (11.0)	1796 (10.3)	375 (16.1)	
Grade, *n* (%)^c^				
Well/moderately differentiated	4926 (24.9)	4352 (25.1)	574 (24.6)	0.4035
Poorly/undifferentiated	14,795 (75.0)	13,037 (74.9)	1758 (75.4)	
Treatment, *n* (%)^c^				
Surgery	10,115 (51.3)	9212 (52.9)	903 (38.7)	<0.0001
Radiation	9336 (47.3)	7964 (45.8)	1372 (58.8)	
Chemotherapy	125 (0.63)	99 (0.57)	26 (1.1)	
No treatment	145 (0.74)	114 (0.66)	31 (1.3)	
# Outpatient visits (mean ± SD)^b^				
Overall	12.2 ± 10.3	12.3 ± 10.4	11.5 ± 9.6	<0.0001
Oncology	9.2 ± 9.0	9.1 ± 8.9	9.3 ± 9.5	<0.0001
Primary care	8.8 ± 12.6	8.6 ± 13.1	9.0 ± 8.7	<0.0001
COC index (mean ± SD)^b^				
Overall	0.44 ± 0.30	0.43 ± 0.29	0.45 ± 0.32	<0.0001
Oncology	0.75 ± 0.29	0.75 ± 0.29	0.77 ± 0.30	<0.0001
Primary care	0.84 ± 0.28	0.84 ± 0.27	0.80 ± 0.30	<0.0001
UPC index (mean ± SD)^b^				
Overall	0.61 ± 0.23	0.61 ± 0.23	0.62 ± 0.23	<0.0001
Oncology	0.84 ± 0.19	0.84 ± 0.19	0.84 ± 0.18	<0.0001
Primary care	0.89 ± 0.18	0.90 ± 0.17	0.88 ± 0.19	<0.0001

Abbreviations: COC, Continuity of Care; SD, standard deviation; UPC, Usual Provider Care.

^a^ *p* for comparison of white and African American prostate cancer patients, with *p* < 0.05 denoting statistical significance.

^b^ *t* Tests for comparison of means.

^c^ Chi square test for comparison of proportions.

### 3.2 Unadjusted comparison of outcomes

As seen from Table 2, in the acute survivorship phase, fewer proportion of African Americans had no ER visits, compared to whites (41.3% vs. 50.8%, *p* value < 0.0001). On the other hand, a larger proportion of African Americans had no hospitalizations, compared to white patients (9.6% vs. 8.7%, *p* value < 0.0001). Compared to whites, the cost of care was higher for African American patients (mean $39,804, SD $39,037 vs. mean $42,639, SD $48,021). Proportion of all‐cause mortality and prostate cancer‐specific mortality was higher among African Americans, compared to white patients (74.9% vs. 61.9%, *p* value < 0.0001; and 37.2% vs. 25.3%, *p* < 0.0001, respectively).

**TABLE 2:** Unadjusted comparison of health service use, cost, and mortality outcomes in the follow‐up period in overall sample, and by race—advanced‐stage prostate cancer.

	All (*N* = 19,721)	White (*n* = 17,389)	AA (*n* = 2332)	*p* value
ER visits, *n* (%)				
0	9800 (49.7)	8838 (50.8)	962 (41.3)	<0.0001
1–3	7206 (36.5)	6286 (36.2)	920 (39.5)	
≥4	2715 (13.8)	2265 (13.0)	450 (19.3)	
Hospitalizations, *n* (%)				
0	1738 (8.8)	1515 (8.7)	233 (9.6)	<0.0001
1–3	10,910 (55.3)	9768 (56.2)	1142 (48.9)	
≥4	7073 (35.9)	6106 (35.1)	967 (41.5)	
Total cost ($), mean ± SD	40,139 ± 40,213	39,804 ± 39,037	42,639 ± 48,021	0.0014
All‐cause mortality, *n* (%)	12,520 (63.5)	10,773 (61.9)	1747 (74.9)	<0.0001
Prostate cancer‐specific mortality, *n* (%)	5262 (6.7)	4395 (25.3)	867 (37.2)	<0.0001

Abbreviations: ER, emergency room; SD, standard deviation.

### 3.3 Multivariable models

For continuity of care (COCI) assessed for all visits, the results from two sets of models for all outcomes are shown in Table 3.

**TABLE 3:** Summary of two series of models on the interactive effects of race and continuity of care (overall) on ER visits, hospitalizations, cost, all‐cause mortality and cancer‐specific mortality, weighted by propensity score—advanced‐stage prostate cancer.

	Model 1: Main effects	Model 2: Model 1 plus interaction
ER visit	IRR (95% CI)	IRR (95% CI)
Race (African American)	1.16 (1.13, 1.18)	1.18 (1.15, 1.22)
COCI score	0.65 (0.64, 0.67)	0.64 (0.63, 0.67)
COCI × African American		0.94 (0.89, 0.98)

Hospitalization	IRR (95% CI)	IRR (95% CI)
Race (African American)	1.12 (1.10, 1.13)	1.16 (1.14, 1.19)
COCI score	0.65 (0.64, 0.67)	0.63 (0.62, 0.64)
COCI × African American		0.89 (0.85, 0.93)

Direct medical care cost	e^*β*^ (95% CI)	e^*β*^ (95% CI)
Race (African American)	1.04 (0.99, 1.08)	1.14 (1.06, 1.21)
COCI score	0.64 (0.61, 0.66)	0.59 (0.56, 0.63)
COCI × African American		0.81 (0.73, 0.91)

All‐cause mortality	HR (95% CI)	HR (95% CI)
Race (African American)	1.30 (1.26, 1.34)	1.64 (1.56, 1.73)
COCI score	0.73 (0.70, 0.75)	0.79 (0.75, 0.82)
COCI × African American		0.46 (0.42, 0.51)

Prostate cancer‐specific mortality	HR (95% CI)	HR (95% CI)
Race (African American)	1.39 (1.32, 1.46)	1.88 (1.74, 2.04)
COCI score	0.78 (0.73, 0.82)	0.88 (0.83, 0.93)
COCI × African American		0.43 (0.37, 0.49)

*Note*: All models were also adjusted for age, marital status, Charlson comorbidity score, grade, and treatment.

Abbreviations: CI, confidence interval; COCI, Continuity of Care Index; e^*β*^, exponent of beta estimate; ER, emergency room; HR, hazard ratio; IRR, incidence rate ratio.

### 3.4 ER visits

We observed that for one unit increase in continuity of care, the percent change in the incident rate of ER visit was 35% lower, after adjusting for socio‐demographic and clinical characteristics. Model 2 showed that the interaction between race and continuity of care (COCI) was statistically significant. For white patients, the percent change in the incident rate of ER visits associated with one unit increase in continuity of care was 36% lower (IRR = 0.64, 95% CI = 0.63, 0.67). The effect of higher continuity of care for African American patients was 0.94 times that for white patients (IRR = 0.94, 95% CI = 0.89, 0.98). The interaction effect indicates by how much the effect of continuity of care differs between groups, that is, between African American and white prostate cancer patients, in multiplicative terms. In summary, the percent change in incident rate of ER visits was 38% lower for African Americans and 36% lower for white patients.

### 3.5 Hospitalizations

The results for model 1 show the main effects of COCI for hospitalizations (IRR, 0.65, 95% CI, 0.64–0.67). Next, model 2 results indicated a statistically significant interaction between race and COCI. The percent change in incident rate of hospitalizations visits associated with one unit increase in continuity of care was 37% lower for white patients (IRR = 0.63, 95% CI = 0.62, 0.64). The effect of higher continuity of care for African Americans was 0.89 times that of their white counterparts (IRR = 0.89, 95% CI = 0.85, 0.93). In summary, the percent change in incident rate of hospitalizations was 42% lower for African Americans and 37% lower for white patients.

### 3.6 Cost

In model 1, we present the main effects of COCI for cost (e^*β*^ = 0.64, 95% CI, 0.61–0.66). In model 2, a statistically significant interaction between race and COCI was observed. For white patients, the reduction in cost associated with one unit increase in continuity of care was 41% (e^*β*^ = 0.59, 95% CI = 0.56, 0.63). The effect of higher continuity of care for African Americans was 0.81 times that of their white counterparts (e^*β*^ = 0.81, 95% CI = 0.73, 0.91). Thus, the percent change in cost was 51% lower for African Americans and 41% lower for white patients.

### 3.7 All‐cause mortality

We present the main effects of COCI for all‐cause mortality (HR, 0.73, 95% CI, 0.70–0.75) in model 1. Results of model 2 indicated a statistically significant interaction between race and COCI. For white prostate cancer patients, the hazard of all‐cause mortality associated with one unit increase in continuity of care was 21% lower (HR = 0.79, 95% CI = 0.75, 0.82). The effect of higher continuity of care for African American prostate cancer patient was 0.46 times that of their white counterparts (HR = 0.46, 95% CI = 0.42, 0.51). Thus, the percent change hazard of all‐cause mortality was 46% lower for African Americans and 21% lower for their white counterparts.

### 3.8 Prostate cancer‐specific mortality

Main effects of COCI for prostate cancer‐specific mortality (HR, 0.78, 95% CI, 0.73–0.82) are shown in model 1. We observed a significant interaction between race and COCI (model 2). For white patients, the hazard of prostate cancer‐specific mortality associated with one unit increase in continuity of care was 12% lower (HR = 0.88, 95% CI = 0.83, 0.93). The effect of higher continuity of care for African American prostate cancer patient was 0.43 times that of white patients (HR = 0.43, 95% CI = 0.37, 0.49). Thus, the percent change in hazard of prostate cancer‐specific mortality was 28% lower for African Americans and 12% lower for white patients.

Our results indicate that the benefits associated with higher continuity of care (COCI) were greater for African Americans, compared to their white counterparts. Comparable results were observed for oncology continuity of care (Table 4) and primary care continuity of care (Table 5). Additionally, results for UPCI for all visits were similar to those for observed for COCI (Tables [Link], [Link]).

**TABLE 4:** Summary of two series of models on the interactive effects of race and continuity of care (oncology) on ER visits, hospitalizations, cost, all‐cause mortality and cancer‐specific mortality, weighted by propensity score—advanced‐stage prostate cancer.

	Model 1: Main effects	Model 2: Model 1 plus interaction
ER visit	IRR (95% CI)	IRR (95% CI)
Race (African American)	1.09 (1.05, 1.12)	1.23 (1.15, 1.31)
COCI score	0.69 (0.66, 0.71)	0.64 (0.62, 0.67)
COCI × African American		0.84 (0.77, 0.91)

Hospitalization	IRR (95% CI)	IRR (95% CI)
Race (African American)	1.02 (1.00, 1.04)	1.13 (1.08, 1.19)
COCI score	0.89 (0.87, 0.90)	0.84 (0.82, 0.87)
COCI × African American		0.86 (0.82, 0.92)

Direct medical care cost	e^*β*^ (95% CI)	e^*β*^ (95% CI)
Race (African American)	0.97 (0.93, 1.02)	1.16 (1.03, 1.31)
COCI score	0.65 (0.62, 0.69)	0.59 (0.56, 0.64)
COCI × African American		0.79 (0.68, 0.92)

All‐cause mortality	HR (95% CI)	HR (95% CI)
Race (African American)	1.25 (1.19, 1.30)	1.77 (1.58, 1.96)
COCI score	0.72 (0.69, 0.76)	0.77 (0.73, 0.81)
COCI × African American		0.49 (0.44, 0.56)

Prostate cancer‐specific	HR (95% CI)	HR (95% CI)
Race (African American)	1.45 (1.36, 1.55)	1.28 (1.09, 1.51)
COCI score	0.61 (0.57, 0.66)	0.59 (0.55, 0.64)
COCI × African American		0.71 (0.59, 0.85)

*Note*: All models were also adjusted for age, marital status, Charlson comorbidity score, grade, and treatment.

Abbreviations: CI, confidence interval; COCI, Continuity of Care Index; e^*β*^, exponent of beta estimate; ER, emergency room; HR, hazard ratio; IRR, incidence rate ratio.

**TABLE 5:** Summary of two series of models on the interactive effects of race and continuity of care (primary care) on ER visits, hospitalizations, cost, all‐cause mortality and cancer‐specific mortality, weighted by propensity score—advanced‐stage prostate cancer.

	Model 1: Main effects	Model 2: Model 1 plus interaction
ER visit	IRR (95% CI)	IRR (95% CI)
Race (African American)	0.98 (0.93, 1.03)	0.96 (0.86, 1.06)
COCI score	0.58 (0.56, 0.62)	0.59 (0.55, 0.63)
COCI × African American		1.04 (0.91, 1.17)

Hospitalization	IRR (95% CI)	IRR (95% CI)
Race (African American)	1.08 (1.04, 1.11)	1.07 (0.99, 1.15)
COCI score	0.71 (0.68, 0.74)	0.71 (0.68, 0.74)
COCI × African American		1.02 (0.92, 1.10)

Direct medical care cost	e^*β*^ (95% CI)	e^*β*^ (95% CI)
Race (African American)	0.98 (0.92, 1.05)	1.12 (0.96, 1.31)
COCI score	0.57 (0.53, 0.61)	0.54 (0.49, 0.59)
COCI × African American		0.85 (0.71, 1.02)

All‐cause mortality	HR (95% CI)	HR (95% CI)
Race (African American)	1.23 (1.14, 1.34)	1.43 (1.18, 1.74)
COCI score	0.69 (0.63, 0.76)	0.72 (0.65, 0.79)
COCI × African American		0.59 (0.48, 0.72)

Prostate cancer‐ specific mortality	HR (95% CI)	HR (95% CI)
Race (African American)	1.28 (1.12, 1.45)	1.85 (1.36, 2.51)
COCI score	0.75 (0.66, 0.87)	0.84 (0.71, 0.97)
COCI × African American		0.52 (0.38, 0.71)

*Note*: All models were also adjusted for age, marital status, Charlson comorbidity score, grade, and treatment.

Abbreviations: CI, confidence interval; COCI, Continuity of Care Index; e^*β*^, exponent of beta estimate; ER, emergency room; HR, hazard ratio; IRR, incidence rate ratio.

### 3.9 Continuity of care in the 2‐year period following the acute survivorship phase

The average COCI for all visits, oncology visits, and primary care visits was comparable to that in the acute survivorship phase. The average COCI for all visits in the 2‐year follow‐up period was 0.48 (SD 0.32) for white patients and 0.51 (SD 0.33) for African American patients. We further observed that the direction of associations between COCI and outcomes in the 2‐year period following acute survivorship phase was comparable to that observed in the acute survivorship phase, albeit of smaller magnitude.

## 4 DISCUSSION

We observed variations in the association between continuity of care and outcomes across African American and white patients. Our two separate measures of continuity of care were COCI (visit dispersion) and UPCI (visit density). We calculated COCI and UPCI for all visits, visits to oncology provider, and visits to primary care provider. Overall, we observed that higher continuity of care was associated with lower incidence rates of ER visits and hospitalizations, and lower cost in the short‐term; and lower mortality (all‐cause and prostate cancer‐specific) in the long‐term (up to 17 years of follow‐up). While both African American and white patients benefited from higher continuity of care, the extent of the benefit was mostly larger for African American patients. Our results have substantial implications for clinical, research (e.g., mechanism via which continuity of care affects process of care including underuse or overuse of medical care; assessing association between other types of continuity [information continuity, and management continuity], process of care and outcomes of care), and policy practices (e.g., capitation or incentive payment system, patient‐centered coordinated care models).

As of year 2021, there were more than 3 million prostate cancer survivors in the United States.^41^ Many prostate cancer patients experience extended survivorship period and thus effective survivorship care is an important element of the prostate cancer care continuum. In this study, we used a large cohort of fee‐for‐service Medicare beneficiaries and observed that higher continuity of care was associated with improved short and long‐term outcomes, and African American patients benefited more than their white counterparts. Research has shown an association between continuity of care and mortality, hospitalization and cost for various diseases and disorders including dementia, diabetes, lung cancer, substance use, and multiple comorbidity.^23^, ^26^, ^27^, ^29^, ^33^, ^34^, ^35^

Providers with different roles can lead to weaker communication and care coordination,^42^ and exacerbate healthcare spending.^16^, ^21^ Given the availability of different treatment options, side effects associated with treatments such as impotence and incontinence, and the prolonged nature of the disease that predisposes the survivors to care fragmentation, continuity of care is crucial for effective management of prostate cancer survivors.^42^

The association between continuity of care for different kinds of visits and outcomes is another key observation made in our study. Studies of survivorship in prostate cancer have shown that engagement of primary care providers in the survivorship care leads to patients receiving improved preventive services and recommended care, compared to patients that were seen by a specialist alone.^43^ In our study, we noted beneficial association between continuity of care for primary visits and mortality; and the magnitude of the benefit was larger for African American patients, compared to white patients. In order to offer necessary coordinated care for prostate cancer survivors, the Institute of Medicine recommended that subspecialists share the treatment summaries and follow‐up plans with the primary care physicians.^43^ Appropriate collaboration among primary care physicians and specialists may aid in refining their individual roles, and consider the unique situations faced by each patient, such as the level of social support and geographic limitations.^15^, ^43^ In addition, African Americans were more likely to experience better outcomes associated with continuity of care with oncology providers than with primary care providers. Given the impaired mortality outcomes relative to white patients and the growing oncologist shortage (especially for survivorship care), efforts are needed, to promote better transitions for African American patients to primary care providers who can provide skilled support and care.

### 4.1 Limitations

Our study has some limitations. We measured COCI and UPCI with the help of outpatient visits that took place in the acute survivorship phase. However, we do not have data regarding the patient‐provider dialog. Our study is observational, and thus we were unable to establish causal association between continuity of care and outcomes. Although we used propensity score to reduce measured biases, some residual bias may persist. Our study cohort comprised fee‐for‐service Medicare beneficiaries of African American or white race, who were at least 66 years of age at the time of prostate cancer diagnosis, were residing in a SEER region and were not enrolled in HMO. In the SEER regions, the distribution of age and race and ethnicity of patients aged 66 years and older is comparable to that of older adults in the general population. However, the SEER regions report a higher proportion of non‐white persons. Also, mortality rates from SEER may not be representative of national cancer mortality rates.^39^ In addition, factors such as comorbidity and insurance status may change over time and affect mortality. However, we did not account for these in our study. We assessed the association between continuity of care and outcomes after adjusting for baseline sociodemographic and clinical characteristics, including comorbidity. Finally, we did not differentiate between planned and unplanned hospitalizations, which may exhibit different association with continuity of care.

In spite of these limitations, our study makes meaningful contribution to the field of continuity of care in advanced prostate cancer patients. In this first of its kind study, we demonstrated the role of continuity of care in acute survivorship phase with short‐term outcomes (ER visits, hospitalizations, and cost); and long‐term mortality (all‐cause and prostate cancer‐specific) in African American and white patients with advanced prostate cancer. Survivorship care planning for prostate cancer must integrate appropriate strategies that will promote continuity of care, especially for patients receiving care from multiple providers. Our future research will focus on analyzing the specific mechanisms via which continuity of care helps alleviate racial disparity and improve the quality for advanced‐stage prostate cancer care.

## AUTHOR CONTRIBUTIONS

**Ravishankar Jayadevappa:** Conceptualization (lead); data curation (lead); formal analysis (supporting); funding acquisition (lead); investigation (lead); methodology (lead); project administration (lead); resources (lead); software (lead); supervision (lead); validation (lead); visualization (lead); writing – original draft (lead); writing – review and editing (lead). **Thomas Guzzo:** Investigation (supporting); writing – original draft (supporting); writing – review and editing (supporting). **Neha Vapiwala:** Investigation (supporting); writing – original draft (supporting); writing – review and editing (supporting). **Stanley Bruce Malkowicz:** Investigation (supporting); writing – original draft (supporting); writing – review and editing (supporting). **Joseph J. Gallo:** Investigation (supporting); methodology (supporting); writing – original draft (supporting); writing – review and editing (supporting). **Sumedha Chhatre:** Conceptualization (equal); data curation (equal); formal analysis (equal); investigation (equal); methodology (equal); validation (equal); writing – original draft (equal); writing – review and editing (equal).

## FUNDING INFORMATION

The study was funded by Agency for Healthcare and Research Quality 1R01HS024106‐01 and the Department of Defense Health Disparity Scholar Award (W81XWH1910461).

## CONFLICT OF INTEREST STATEMENT

None of the authors has conflict of interest to report.

## Supporting information


Figure S1.

Table S1.

Table S2.

Table S3.

Table S4.

Table S5.

## Data Availability

Data sharing not applicable—no new data generated.
