# Supplementary figures and images for: Continuity of care and advanced prostate cancer
Source: Cancer Med. 2023 Mar 23;12(10):11795–805. doi: 10.1002/cam4.5845 (PMC10242338; doi:10.1002/cam4.5845)

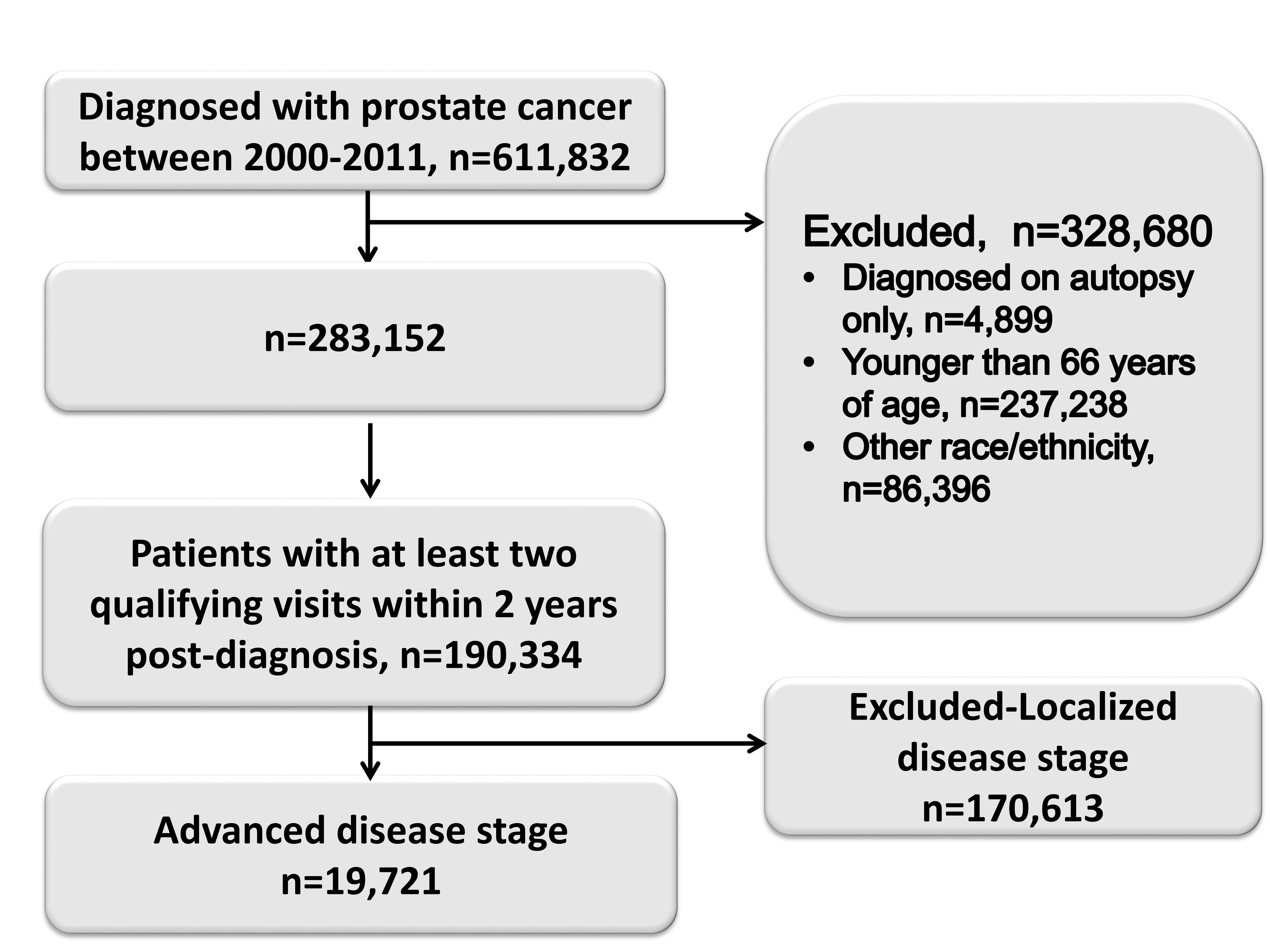

Supplement: Supplementary file 1 — Figure S1. [file CAM4-12-11795-s001.jpg]
