# Supplementary material for: Continuity of care and advanced prostate cancer
Source: Cancer Med. 2023 Mar 23;12(10):11795–805. doi: 10.1002/cam4.5845 (PMC10242338; doi:10.1002/cam4.5845)
Supplement: Supplementary file 2 — Table S1. [file CAM4-12-11795-s005.docx]

Supple Table 1. Common Procedure Terminology codes for outpatient visits

| 99201-99205, 99211-99215, 99241-99245, 99251-99255, 99304-99310, 99324-99328, 99334-99337, 99341-99345, 99347-99350, 99354-99355, 99358-99359, 99381-99387, 99391-99397, 99401-99404, 99406-99409, 99411-99412, G0402, G0438, and G0439 |
| --- |
