# Supplementary material for: Continuity of care and advanced prostate cancer
Source: Cancer Med. 2023 Mar 23;12(10):11795–805. doi: 10.1002/cam4.5845 (PMC10242338; doi:10.1002/cam4.5845)
Supplement: Supplementary file 3 — Table S2. [file CAM4-12-11795-s004.docx]

Supple Table 2. Physician Specialty Codes (American Medical Association)

| **Oncology** | **Primary care** |
| --- | --- |
| - Anesthesiology - Colon & Rectal Surgery - Diagnostic Radiology - Hematology/Oncology - Medical Oncology - Radiation Oncology - Radiology - Surgical Oncology - Urology | - Emergency Medicine - Emergency Medicine/Family Medicine - Epidemiology - Family Medicine - General Practice - General Preventive Medicine - Geriatric Medicine (Family Medicine) - Geriatric Medicine (Internal Medicine) - Geriatric Psychiatry - Hematology (Internal Medicine) - Internal Medicine - Internal Medicine/Family Medicine - Internal Medicine/Preventive Medicine - Physical Medicine & Rehabilitation - Public Health and General Preventive Medicine - Urgent Care Medicine |
