# Supplementary material for: Continuity of care and advanced prostate cancer
Source: Cancer Med. 2023 Mar 23;12(10):11795–805. doi: 10.1002/cam4.5845 (PMC10242338; doi:10.1002/cam4.5845)
Supplement: Supplementary file 4 — Table S3. [file CAM4-12-11795-s003.docx]

**Supple Table 3.** Summary of 2 Series of Models on the Interactive Effects of Race and Continuity of Care (overall UPC) on ER visits, hospitalizations, cost, all-cause mortality and cancer-specific mortality, weighted by propensity score* – advanced stage.

|  | **Model 1: Main Effects** | **Model 2: Model 1 Plus Interaction** |
| --- | --- | --- |
| **ER visit** | **IRR (95% CI)^**^** | **IRR (95% CI)^**^** |
| Race (African American) | 1.16 (1.12, 1.19) | 1.14 (1.06, 1.23) |
| UPC score | 0.51 (0.48, 0.53) | 0.51 (0.48, 0.54) |
| UPC x African American |  | 1.03 (0.92, 1.16) |
|  | | |
| **Hospitalization** | **IRR (95% CI)^**^** | **IRR (95% CI)^**^** |
| Race (African American) | 1.12 (1.09, 1.14) | 1.16 (1.09, 1.22) |
| UPC score | 0.51 (0.49, 0.53) | 0.50 (0.48, 0.52) |
| UPC x African American |  | 0.93 (0.85, 1.02) |
|  | | |
| **Direct Medical Care Cost** | **e^β^ (95% CI) ^***^** | **e^β^ (95% CI)^***^** |
| Race (African American) | 1.04 (0.99, 1.08) | 1.22 (1.11, 1.35) |
| UPC score | 0.52 (0.49, 0.54) | 0.47 (0.44, 0.50) |
| UPC x African American |  | 0.76 (0.66, 0.88) |
|  | | |
| **All-cause Mortality** | **HR (95% CI)^&^** | **HR (95% CI)^&^** |
| Race (African American) | 1.29 (1.23, 1.37) | 1.92 (1.68, 2.19) |
| UPC score | 0.75 (0.69, 0.80) | 0.82 (0.76, 0.89) |
| UPC x African American |  | 0.43 (0.36, 0.52) |
|  | | |
| **Prostate Cancer-specific Mortality** | **HR (95% CI)^&^** | **HR (95% CI)^&^** |
| Race (African American) | 1.39 (1.29, 1.51) | 2.36 (1.91, 2.92) |
| UPC score | 0.78 (0.70, 0.88) | 0.91 (0.80, 1.02) |
| UPC x African American |  | 0.38 (0.28, 0.51) |

* All models were also adjusted for age, marital status, Charlson comorbidity score, grade and treatment.

** IRR = Incidence rate ratio

*** e^β^  = exponent of beta estimate

& HR = Hazard ratio
