# Supplementary material for: Continuity of care and advanced prostate cancer
Source: Cancer Med. 2023 Mar 23;12(10):11795–805. doi: 10.1002/cam4.5845 (PMC10242338; doi:10.1002/cam4.5845)
Supplement: Supplementary file 5 — Table S4. [file CAM4-12-11795-s002.docx]

**Supple Table 4.** Summary of 2 Series of Models on the Interactive Effects of Race and Continuity of Care (oncology UPC) on ER visits, hospitalizations, cost, all-cause mortality and cancer-specific mortality, weighted by propensity score* – advanced stage.

|  | **Model 1: Main Effects** | **Model 2: Model 1 Plus Interaction** |
| --- | --- | --- |
| **ER visit** | **IRR (95% CI)**** | **IRR (95% CI)**** |
| Race (African American) | 1.25 (1.18, 1.33) | 1.41 (1.12, 1.79) |
| UPC score | 0.53 (0.48, 0.58) | 0.54 (0.48, 0.59) |
| UPC x African American |  | 0.46 (0.36, 0.60) |
|  | | |
| **Hospitalization** | **IRR (95% CI)**** | **IRR (95% CI)**** |
| Race (African American) | 0.78 (0.71, 0.85) | 0.46 (0.31, 0.69) |
| UPC score | 0.39 (0.34, 0.44) | 0.36 (0.32, 0.42) |
| UPCI x African American |  | 0.68 (0.43, 1.07) |
|  | | |
| **Direct Medical Care Cost** | **e^β^ (95% CI) ^***^** | **e^β^ (95% CI)^***^** |
| Race (African American) | 1.12 (1.08, 1.16) | 1.28 (1.19, 1.48) |
| UPC score | 0.69 (0.65, 0.73) | 0.65 (0.59, 0.71) |
| UPC x African American |  | 0.84 (0.71, 0.99) |
|  | | |
| **All-cause Mortality** | **HR (95% CI)^&^** | **HR (95% CI)^&^** |
| Race (African American) | 1.09 (1.05, 1.11) | 1.44 (1.29, 1.61) |
| UPC score | 0.89 (0.86, 0.93) | 0.78 (0.74, 0.84) |
| UPCI x African American |  | 0.71 (0.62, 0.80) |
|  | | |
| **Prostate Cancer- specific** | **HR (95% CI)^&^** | **HR (95% CI)^&^** |
| Race (African American) | 1.22 (1.18, 1.29) | 2.61 (2.13, 3.18) |
| UPC score | 0.57 (0.54, 0.61) | 0.39 (0.35, 0.45) |
| UPC x African American |  | 0.40 (0.32, 0.51) |

* All models were also adjusted for age, marital status, Charlson comorbidity score, grade and treatment.

** IRR = Incidence rate ratio

*** eβ = exponent of beta estimate

& HR = Hazard ratio
