# Supplementary material for: Continuity of care and advanced prostate cancer
Source: Cancer Med. 2023 Mar 23;12(10):11795–805. doi: 10.1002/cam4.5845 (PMC10242338; doi:10.1002/cam4.5845)
Supplement: Supplementary file 6 — Table S5. [file CAM4-12-11795-s006.docx]

**Supple Table 5.** Summary of 2 Series of Models on the Interactive Effects of Race and Continuity of Care (primary care UPC) on ER visits, hospitalizations, cost, all-cause mortality and cancer-specific mortality, weighted by propensity score* – advanced stage.

|  | **Model 1: Main Effects** | **Model 2: Model 1 Plus Interaction** |
| --- | --- | --- |
|  |  |  |
| **ER visit** | **IRR** (95% CI)** | **IRR** (95% CI)** |
| Race (African American) | 1.31 (1.19, 1.45) | 0.66 (0.45, 0.97) |
| UPC score | 0.79 (0.66, 0.96) | 0.66 (0.53, 0.82) |
| UPC x African American |  | 1.50 (1.01, 2.25) |
|  | | |
| **Hospitalization** | **IRR** (95% CI)** | **IRR** (95% CI)** |
| Race (African American) | 0.63 (0.53, 0.74) | 0.29 (0.15, 0.54) |
| UPC score | 0.88 (0.66, 1.17) | 0.75 (0.55, 1.02) |
| UPC x African American |  | 1.91 (0.98, 3.70) |
|  |  |  |
| **Direct Medical Care Cost** | **e^β^ (95% CI) ^***^** | **e^β^ (95% CI)^***^** |
| Race (African American) | 1.02 (0.96, 1.09) | 1.78 (1.45, 2.19) |
| UPC score | 0.55 (0.49, 0.62) | 0.46 (0.41, 0.52) |
| UPC x African American |  | 0.50 (0.39, 0.64) |
|  |  |  |
| **All-cause Mortality** | **HR (95% CI)^&^** | **HR (95% CI)^&^** |
| Race (African American) | 1.01 (0.96, 1.06) | 1.04 (0.87, 1.22) |
| UPC score | 0.56 (0.52, 0.61) | 0.56 (0.51, 0.62) |
| UPC x African American |  | 0.97 (0.80, 1.16) |
|  | | |
| **Prostate cancer- specific mortality** | **HR (95% CI)^&^** | **HR (95% CI)^&^** |
| Race (African American) | 0.95 (0.90, 1.00) | 1.46 (1.17, 1.81) |
| UPC score | 0.43 (0.39, 0.47) | 0.36 (0.32, 0.41) |
| UPC x African American |  | 0.60 (0.47, 0.77) |

* All models were also adjusted for age, marital status, Charlson comorbidity score, grade and treatment.

** IRR = Incidence rate ratio

*** e^β^  = exponent of beta estimate

& HR = Hazard ratio
